# Editorial: Prostate cancer research: tools, cancer cell types, molecular targets

**DOI:** 10.3389/fonc.2025.1561382

**Published:** 2025-02-04

**Authors:** Hung-Ming Lam, Alvin Y. Liu

**Affiliations:** Department of Urology, University of Washington School of Medicine, Seattle, WA, United States

**Keywords:** prostate cancer, noncoding RNA (ncRNA), bone metastasis, tumor microenvironment (TME), patient-derived xenografts (PDX), TP53 polymorphism, immunohistochemistry, tumor immune evasion

Prostate cancer (PCa) is a heterogeneous disease and research tools have become more available now than ever. In this Research Topic, we organized a collection of reviews and a research report that discussed the tumor-intrinsic and -extrinsic events of PCa, and tools available for research.

Sang et al. summarized the literature on the involvement of noncoding RNA (ncRNA) in PCa bone metastasis. In this review, three species of ncRNA, micro (miRNA), long non-coding (lncRNA), and circular (circRNA), were discussed with respect to the signaling pathways of NF-κB, WNT/β-catenin, TGF-β, PI3K/AKT, MMP-chemokine; the specific gene members targeted by the differentially expressed RNA in tumors that promote or inhibit bone metastasis. The interaction between cancer cells and cells in the bone microenvironment is likely via secreted exosomes containing these RNA. There are well-established patient-derived xenografts (PDX) from bone metastases, LuCaP 77 and LuCaP 105, and intra-tibial models (1) for mechanistic investigation of ncRNA in bone metastasis. The PDX cells can be grown in mouse bone causing osteoblastic, osteolytic or mixed lesions. Most patients from whom the PDX lines were established had undergone treatment, and showed castration-resistant PCa. Patient survival in this donor group was between <1 to ~15 years, with different Gleason scores in their primary tumors. A study method is described in Tools to adapt the xenografts to *in vitro* growth and long term storage so that these cells can be readily studied in the lab such as differential expression analysis of ncRNA between bone- and non-bone-derived lines, transfection of ncRNA to see their effect on targeted genes, or whether certain species could inhibit growth in bone and those that allow cancer cells to grow in bone. With respect to the outline of lineage relationship among cancer cell types in Tools, what are the differentially expressed ncRNA between luminal-like adenocarcinoma and stem-like small cell carcinoma? Could the pattern change when small cell carcinoma are induced by stromal factor PENK to undergo differentiation, or when adenocarcinoma are reprogrammed by stem cell transcription factors (scTF)?

Toscano-Guerra et al. reported the association of TP53 rs1042522 single nucleotide polymorphism (SNP) with PCa risk in a Spanish Caucasian population. TP53 is a tumor suppressor gene frequently mutated in cancer, and the rs1042522 SNP (also known as P72R polymorphism) may influence PCa susceptibility. Reports have shown conflicting results on P72R and PCa risk, and aggressiveness (2–5). This study reported that P72R was associated with PCa risk in the following: (1) aggressive high-grade primary PCa cultures (n=12, Gleason ≥ 8), (2) localized and lower grade PCa from prostatectomy (n=11, Gleason ≤7), (3) serum from patients (n=94). The association between P72R and PCa risk was particularly significant with serum, with an odds ratio of 7.937 (95% CI 5.37-11.00), highlighting the potential of P72R in identification of men at risk for PCa. This study also discussed the importance of selecting appropriate population controls. For example, P72R is detected in 29% of control European population but 67% of control African population from the 1000 Genomes Project, demonstrating the importance of race or ethnicity.

Tumor microenvironment (TME) plays a key role in how tumors respond to therapies, and potential mechanisms of treatment resistance. San-Jose Manso et al. reviewed the immunome (cells of the immune system) of PCa, which is characterized as an immunologically “cold” tumor due to low T-cell response to the cancer. Tumor immune evasion is enhanced by decreases in effector T cells and increases in regulatory T cells. How tumor cells affect cells of the immune system is largely unknown. The CD profiles of prostate (as described in Tools) showed a sizable population of infiltrating CD45+ white blood cells. Over 70 of the CD specificities tested could be detected by immunohistochemistry, including CD3 (T cell activating cells), CD4 (helper T), CD6, CD8 (cytotoxic T), CD11b, CD11c (dendritic cells), CD13, CD14 (myelomonocytic cell), CD18, CD27, CD32, CD38, CD43, CD44, CD53, CD66b (granulocyte), CD68 (macrophage), CD69 (maturing NK cell), CD74, CDw78, CD83, CD93, CD117, CD161 (activating NK cell), CD162. All these commercially available antibodies could be used to identify differential expression of immune system cells by immunohistochemistry among normal/benign prostate, PCa of Gleason patterns 3, 4, 5, as well as local and distant metastases. The effective antibody titers for immunohistochemistry of frozen sections have been reported (6). The immunome review highlighted PCa exhibiting an immunosuppressive TME mainly comprised of MDSC, TAM, and different soluble factors (TGF, IL-6/10/23), frequently leading to immunological tolerance of the tumor. As such, new strategies to overcome this suppressed immune system in PCa will be clinically important.

For studies on cell-to-cell interaction (as described in Tools), specific lymphocytes, e.g., CD4+, CD8+, CD45+, from resected normal/benign *vs*. tumor tissue, or from donor blood samples are co-cultured with CD26+ cancer cells; CD90+ cancer-associated stromal cells (CPstrom) *vs*. CD49a+ stromal cells (NPstrom). As a substitute, the many LuCaP lines representing adenocarcinoma, non-adenocarcinoma, and small cell carcinoma can be used. The cell types are cultured with or without cell contact, and monitored by transcriptomics and cell appearance. The co-culture is used to demonstrate (1) possible immunosuppressive effect of cancer cells; (2) effect of CPstrom (e.g., via stromal-derived growth factor-1, monocyte chemotactic protein) in altering the gene expression of immune cells (e.g., macrophages), which may enhance tumor cell behavior. CPstrom also differ from NPstrom in chemokine expression of many CXCL molecules.

The immunome review also reports on systemic immune response to treatments such as androgen deprivation, which may stimulate CD4+, CD8+ T cells, and macrophages to promote androgen production; androgen receptor signaling inhibition, which could lead to expansion of monocytes; chemotherapy with docetaxel and radiotherapy, which could lead to a pro-inflammatory cascade; checkpoint blockade inhibition with hypoxia, which could induce increases in PDL-1. These reported investigations into the body’s immune response post treatment are attempts to explain success or failure of these modalities. Some examples include activation of dendritic cells (plasma cytoid, classical subtypes), myeloid-derived suppressor cells to activate neutrophils, monocytes with inhibition of T cells, B cells, NK cells, CXCL5/CXCR2, IL23 signaling, and other soluble immune related factors – IL-6, TGF-β, IL-10 with correlation to Gleason grades, TNF-α, INF-γ with increased tumor cell apoptosis. Appropriate *in vitro* studies can be designed to test these observations with the responsible immune cell types.

With our improved understanding on PCa, as well as tools for studying tumor and TME interaction, the field is now better equipped to improve early detection and develop novel treatment strategies to enhance patient outcome.
